# Investigating Apoptotic Effects of Methanolic Extract of *Dorema glabrum* Seed on WEHI-164 Cells

**DOI:** 10.1155/2013/949871

**Published:** 2013-07-17

**Authors:** Maryam Bannazadeh Amirkhiz, Nadereh Rashtchizadeh, Hossein Nazemiyeh, Jalal Abdolalizadeh, Leila Mohammadnejad, Behzad Baradaran

**Affiliations:** ^1^Drug Applied Research Center, Tabriz University of Medical Sciences, Tabriz 5165665811, Iran; ^2^Tabriz University of Medical Sciences, International Branch (Aras), Jolfa 5441643177, Iran; ^3^Research Center for Pharmaceutical Nanotechnology, Tabriz University of Medical Sciences, Tabriz 5165665811, Iran; ^4^Immunology Research Center, Tabriz University of Medical Sciences, Tabriz 5165665811, Iran

## Abstract

We aimed to investigate the apoptotic effects of the methanolic extract of *Dorema glabrum* seed on WEHI-164, cancerous cells in comparison with L929, normal cells and compared them with the cytotoxic effects of Taxol. So, MTT test and DNA fragmentation assay were performed on cultured and treated cells. Also electrophoresis which was followed by immunoblotting was done to survey the production of Caspase-3 and Bcl_2_ proteins, and to inquire into their relative genes expression, RT-PCR was used. According to our findings, the methanolic extract of *Dorema glabrum* seed can alter cells morphology as they shrink and take a spherical shape and lose their attachment too. So, the plant extract inhibits cell growth albeit in a time- and dose-dependent manner and results in degradation of chromosomal DNA. Induction of apoptosis by the plant extract was proved by the reduction of pro-Caspase-3 and Bcl_2_ proteins and increase in Caspase-3 gene expression and decrease in that of bcl_2_ too. Our data well established the antiproliferative effect of methanolic extract of *Dorema glabrum* seed and clearly showed that the plant extract can induce apoptosis and not necrosis in vitro. These results demonstrated that *Dorema glabrum* seed might be a novel and attractive therapeutic candidate for tumor treatment.

## 1. Introduction

Normal cells grow and divide in an ordered fashion, in accordance with the cell cycle. Defective apoptosis (programmed cell death) which results in enhanced growth describes most cancer cells [1]. Several proteins control the timing of the events in the cell cycle, which is tightly regulated to ensure that cells divide only when necessary. The loss of this regulation is the hallmark of cancer [1, 2]. Initially, somatic cell fusion and nuclear transplantation studies, together with the selective use of growth factors and inhibitors of macromolecular biosynthesis, established the fundamental parameters of cell cycle regulation [3, 4]. Our understanding of the complexities of apoptosis and the mechanisms evolved by tumor cells to resist engagement of cell death has focused research effort into the development of strategies designed to selectively induce apoptosis in cancer cells [5–7].

Several previous studies demonstrated that certain phytochemicals present in medicinal herbs exert antitumorigenic activity by inducing apoptosis in cancer cells [8–11]. The mechanism, of apoptosis are now mostly well known, involving activation of caspases (cysteinyl, aspartate-specific proteases), which cleave to inactivate or activate target substrates within a cell [5] and Bcl_2_ family members in response to a wide variety of physiological and injury-induced signals [12].

Caspases are synthesized in most if not all cells as inactive zymogens, which must be proteolytically cleaved at two (or three in some cases) aspartate residues to generate the active mature enzyme. The generation of active caspases forms a cascade in which “initiator” caspases interact with specific adapter molecules to facilitate their own autoprocessing. These now active initiator caspases notably Caspase-8 or FLICE, being “apical” and more susceptible to modification by endogenous regulatory proteins, in turn cleave and activate the downstream “executioner” caspases, such as Caspase-3 also known as apopain, SCA-1, Yama, and CPP32 (Alias), which enact the final, irreversible commitment to death [13]. These then cleave their target substrates to orchestrate the proteolytic dismantling of the cell [14, 15]. This sequence of events culminating in the activation of caspases has been broadly categorized into two pathways, the “extrinsic” pathway characterized by the engagement of cell surface “death receptors” and the “intrinsic” pathway involving key mitochondrial events [5, 15].

The Bcl_2_ proteins also represent a promising target for modulating tumor cell sensitivity to apoptosis [5, 16]. It was first human apoptotic protein, an inhibitor of apoptosis identified in 1984 [17, 18]. High amounts of Bcl_2_ block the apoptotic death of a pro-B-lymphocyte cell line. Thus, Bcl_2_ is unique among protooncogenes, being localized to mitochondria and interfering with programmed cell death independent of promoting cell division [19]. Overexpression of antiapoptotic Bcl_2_ proteins is observed in many tumor types, which may contribute to the drug-resistant state and help mediate the expansion of a transformed population by disrupting normal cell turnover [5, 20, 21].

Chemotherapy drugs are toxic compounds that target rapidly growing cells. So, these drugs can also eliminate certain adult cells that divide more rapidly, such as those that line the gastrointestinal tract, bone marrow cells, and hair follicles. This causes some side effects, including gastrointestinal distress, low white blood cell count, and hair loss [22–24].

For several millennia, herbal preparations and natural remedies have been shown to be effective in treating many types of maladies [9, 25, 26]. Although herbal therapies are becoming increasingly popular worldwide, we know little about the molecular mechanisms and active ingredients in many of those therapeutic herbs [9, 27]. Chemical characterization and cytotoxicity screening studies on plant-based materials could lead to a discovery of new natural anticancer drugs [28].

Iran has unique plant varieties yet to be studied for anticancer components. Just to mention, Valiyari et al. studied apoptosis-inducing properties of dichloromethanoic and methanolic extracts of *Scrophularia oxysepala* in MCF-7 human breast cancer cells [8], and Samavati et al. investigated antitumoral effects of *Ornithogalum cuspidatum* on WEHI-164 cells [29].

*Dorema glabrum*, a medicinal plant of the family of Apiaceae, which grows in Transcaucasia (Nakhichevan and Armenia zones) and north west of Iran (Azerbaijan), in loamy or rocky slopes of Aras river, was chosen to study its apoptotic effects on mouse fibrosarcoma cell line, WEHI-164 cells in comparison with its effects on L929 mouse normal cells. The plant has extensive uses, for example, as an herbal remedy or food additive in the mentioned regions [30]. This study was conducted according to the common folk beliefs of Armenian and Azeri people that *Dorema glabrum* can cure many anomalies especially different kinds of cancer. Of course it should be mentioned that in a preliminary work, the crude extract of the plant demonstrated antioxidant activity and antilipidemic effects [31].

## 2. Materials and Methods

### 2.1. Plant Material

Seeds of *Dorema glabrum* Fisch. C.A. Mey were collected during the fruiting stage from rocky slopes of Aras River bank; Jolfa, Eastern Azerbaijan (38 30′9.2′′, 45 27′36.2′′; 1590 m, 15 km from Jolfa to St. Stephanus Church), Iran. Air-dried and finely powdered seeds were subjected to extraction by refluxing methanol in a soxhlet in order to obtain its ooze. Then, the extract was dried using a Rotary Evaporator. 20 mg of dried extract was dissolved in 100 *μ*L DMSO and diluted with 3.90 mL RPMI-1640 to give a concentration of 5000 *μ*g/mL. This was used to treat the cells.

### 2.2. Cell Culture

WEHI-164 cells, mouse fibrosarcoma cell line (NCBI code C200) and L929 cells, mouse normal adipose tissue cell line (NCBI code C161) were obtained from Iran Pasture institute. Both cell lines were cultured in RPMI-1640 (Sigma, pH = 7.2) containing 10% FCS (fetal calf serum) and antibiotic (100 U/mL penicillin, 100 *μ*g/mL streptomycin), placed in 37°C and 5% CO_2_ in an incubator overnight.

### 2.3. MTT Test

MTT assay is one of the most useful tests for investigating cells viability and cytotoxic effects of drugs, cosmetics, and food additives. MTT (3-[4, 5-dimethyl-2-thiazolyl]-2, 5 diphenyl tetrazolium bromide), which is yellow and soluble in water, can be reduced by mitochondrial dehydrogenases of live cells to give a bluish purple and insoluble salt called Formosan that can easily and rapidly be quantitated by an ELISA plate reader at 570 nm to evaluate growth and viability of cells and cytotoxic effects of interventor agents [13, 32].

WEHI-164 and L929 cells were separately seeded, in a triplicate manner, in 96-well microplates (5000 cells/well) as above. After 6 hours, both cells were treated with different concentrations (10, 30, 50, 100, 200, 300, and 400 *μ*g/mL) of methanolic extract of *Dorema glabrum *seeds with different time periods (6, 24, and 36 hours). No plant extract was added to negative controls, but the same amount of DMSO was added to eliminate its intervening effects, if any. Positive control cells were treated with Taxol as the same concentrations of plant extract in test cells. Taxol which contains paclitaxel as the main active compound is used in chemotherapy of cancer. Of course prior to treatment, the cells viability was determined by counting on a Neubauer slide (Hemocytometer) with the aid of Trypan blue. Trypan blue can penetrate into dead cells' membrane and colour them purple.

After desired time, the supernatants of all wells were discarded and washed with PBS; then 100 *μ*L of RPMI and 50 *μ*L of MTT solution (2 mg/mL) were added to each well. Following incubation at 37°C for 4 hours, the liquid phase of wells was discarded again. After adding 200 *μ*L DMSO and 25 *μ*L Sorensen's glycine buffer (0.1 M glycine, 0.1 M NaCl; pH = 10.5), the plates were incubated at 37°C in the dark for another half an hour. At the end, absorbencies of wells were determined at 570 nm wavelength using a microplate reader (Awareness Technology, USA).

### 2.4. Electrophoresis and Immunoblotting

To survey production of proteins involved in apoptosis, SDS-PAGE and immunoblotting were performed. Electrophoresis and related applications have contributed greatly to the understanding of the molecular bases of cell structure and function. To enhance resolution and discrimination of proteins on the basis of molecular size rather than charge or shape, the proteins were denatured by SDS, prior to electrophoresis [33].

The western blot is a widely accepted analytical technique used to detect specific proteins in the given sample. Proteins separated by electrophoretic technique are electrophoretically transferred (“electroblotted”) onto a membrane. The membrane, which is now a replica of the polyacrylamide gel, is subsequently probed with antibodies to specific proteins. The primary antibodies can be revealed by an additional incubation with HRP conjugated, followed by enhanced electrochemiluminescent (ECL) detection [33].

#### 2.4.1. Protein Extraction

Prior to electrophoresis, we need to extract cells' proteins; hence WEHI-164 cells were cultured in 6-well plates (10^6^ cells per well) with RPMI 1640 containing 10% FCS and antibiotic (total volume of 5 mL). Six hours later, treatments with different concentrations of plant extract (0, 30, 50, and 100 *μ*g/mL) with different time periods (24 and 36 hours) were done. According to MTT results and IC_50_ value of the plant extract, there was no need to treat cells with concentrations more than 100 *μ*g/mL. The wells with no extract considered as negative controls, and to eliminate effects of DMSO the same amount of DMSO concentrations in test cells were added to them. And as MTT test, positive control cells were treated with Taxol as the same concentrations of extract in test cells.

Twenty-four and 36 hours after treatment, the supernatants of all wells were gathered and kept in separate tubes. This time we did not discard the supernatants because we needed the dead cells that were affected by plant ooze to extract their proteins. All wells were washed with PBS and cells were detached from bottom of plates' wells using Trp-EDTA, and all liquids and cells from each well was added to its own supernatant. The tubes were centrifuged at 2000 RPM for 10 min and then washed two times with PBS at 2500 RPM for 5 min. The cells pellets were homogenized by gentle vortexing and removed to microtubes with 200 *μ*L of PBS and centrifuged at 1500 RPM for 5 min. The supernatants were discarded and pellets were completely homogenized by gentle vortexing. 200 *μ*L of lysis buffer was added to each tube and following mixing gently, left on the rotator in 4°C for half an hour. The tubes were centrifuged again at 15000 RPM for 15 min. Then, supernatants were removed to fresh microtubes and pellets were discarded. To obtain protein concentration of each sample, absorbance of each tube, at 280 nm wavelength, was read using nanodrop spectrophotometer against lysis buffer blank.

#### 2.4.2. SDS-PAGE and Electroblotting

As mentioned above, to investigate production of proteins involved in apoptosis, SDS-PAGE and western blotting should be performed. SDS-PAGE was performed under reducing conditions using 12.5% polyacrylamide slab gels. Then, proteins on the gel were transferred to the PVDF membrane using a semidry electroblotting tank. Then, the membrane was washed three times (every time 5 minutes) with wash buffer (0.05% Tween 20 in PBS) and was soaked in the primary antibody (Abcam, USA) with a dilution of 1/1000 on the shaker for two hours, followed by again three times washing with wash buffer. 0.03% of HRP conjugated secondary antibody (Abcam, USA, 3 *μ*L in 10 mL wash buffer, at 1/5000 dilution) was used to incubate the membrane on the shaker, away from direct light, for 2 hours. After discarding the secondary antibody also the membrane was washed three times with wash buffer. The protein bands in the membrane were developed using ECL substrate on radiography film.

### 2.5. Investigating bcl_**2**_ and Caspase-3 Genes Expression

In order to investigate expression of bcl_2_ and Caspase-3 genes, to confirm Western blotting results, RT-PCR was performed. Hence, PCR was performed on synthesized cDNAs, using WEHI-164 cells' mRNA. 

#### 2.5.1. RNA Extraction

The cells were cultured and treated as mentioned above. Since mRNA is the first thing to be synthesized in genes expression and also mRNAs are very unstable, briefly (3 and 6 hours) treated cells were used as well. After detaching cells from the plates and doing the washing process with PBS, their precipitants were transferred to RNase-free microtubes. Total RNA was extracted using RNX-Plus solutions according to the manufacturer's protocol, and because RNA is very unstable, work always should be done on ice. Purified RNAs were dried and then dissolved in 50 *μ*L DEPC-water. Then, cDNAs were transcribed from RNAs as described below.

#### 2.5.2. cDNA Synthesis

Prior to cDNA synthesis, RNA contents of samples were determined using a nanodrop UV spectrophotometer. Then keeping in mind the samples concentrations, the desired volume (for 20 *μ*L reaction a volume equivalent to 3 *μ*g RNA) of each sample was added to reaction mixture, containing 100 pmole oligo dt in a sterile RNase-free tube. Then, the mixtures were incubated at 70°C for 5 minutes. After transferring the mixtures to AccuPower RT PreMix tubes, they filled up to 20 *μ*L with DEPC distilled water. The mixtures were vortexed to dissolve the pellets and briefly spun down. To avoid samples evaporation, one drop of mineral oil was added to each sample before putting in thermal cycler apparatus. The strips were put in the thermal cycler apparatus under the following conditions: cDNA synthesis at 42°C for 60 minutes and 94°C for 5 minutes to inactivate RTase and terminate the reaction.

#### 2.5.3. cDNA PCR

The volume of each reaction was considered to be 20 *μ*L. The Ampliqon master mix that is 2X was used to make reaction mixture as follows: 2 *μ*L of each cDNA sample was added to 10 *μ*L of master mix then 1 *μ*L of each forward and reverse primers (10 mM) and 6 *μ*L of distilled, deionised, sterile water were added to make the final volume of 20 *μ*L and MgCl_2_ concentrations of 1.5 mM. In the end, one drop of mineral oil was added to the top of each sample to avoid evaporation of samples.

Then microtubes were put in the thermal cycler apparatus under the following conditions: initial denaturation at 94°C for 4 minutes, followed by 35 amplification cycles, each consisting of denaturation at 94°C for 30 seconds, annealing at 58°C for 30 seconds, and extension at 72°C for 30 seconds, with an additional extension step at the end of the procedure at 72°C for 5 minutes.

All RT-PCR products were visualized by electrophoresis through 2% agarose gel followed by ethidium bromide staining.

### 2.6. DNA Fragmentation Assay

The biochemical hallmark of apoptosis is the fragmentation of the genomic DNA, an irreversible event that commits the cell to die and occurs before changes in plasma membrane permeability (prelytic DNA fragmentation). The DNA laddering technique is used to visualize the endonuclease cleavage products of apoptosis. This assay involves extraction of DNA from a lysed cell homogenate followed by agarose gel electrophoresis. 

#### 2.6.1. DNA Extraction

After culturing and treating cells with different concentrations of plant extract (0, 30, 50, and 100 *μ*g/mL) for different time periods (24 and 36 hours), their genomic DNA contents were extracted by firstly lysing the cells using 500 *μ*L of lysis buffer. Then 10 *μ*L of proteinase K (Fermentas, Life Sciences, 20 mg/mL) were added and the samples were incubated at 56°C overnight. Next day, 40 *μ*L of saturated NaCl (5 M) was added and mixed completely, followed by incubation at 4°C for 10 minutes. After 20-minute centrifugation in 12000 RPM, their upper liquids were transferred to a fresh microtube. 1 mL of cold ethanol 100% (stored in −20°C) was added and then incubated at −20°C for 10 minutes. After centrifugation for 15 minutes in 12000 RPM, the ethanol in upper phase was removed completely. Following adding 1 mL of ethanol 70 (kept in 4°C) and centrifugation for 10 minutes in 12000 RPM, the ethanol was removed completely again. The samples were left at room temperature or 37°C for 10–20 minutes to dry. The pellets were dissolved in 100 *μ*L distilled, deionized, and sterile water or TE (Tris/EDTA).

#### 2.6.2. Agarose Electrophoresis

After determining the samples concentrations using a nanodrop UV spectrophotometer, the equivalent amount of DNA samples diluted with the 6X DNA loading dye (supplied with the ladder) were subjected to 1.5% agarose submarine electrophoresis in company with DNA ladder marker (Fermentas, Life Sciences, 1 kb DNA Ladder). In the end, following ethidium bromide staining, the fragmented DNAs bands were visualized by UV transilluminator.

## 3. Results

### 3.1. Cell Culture

**Figure 1:**
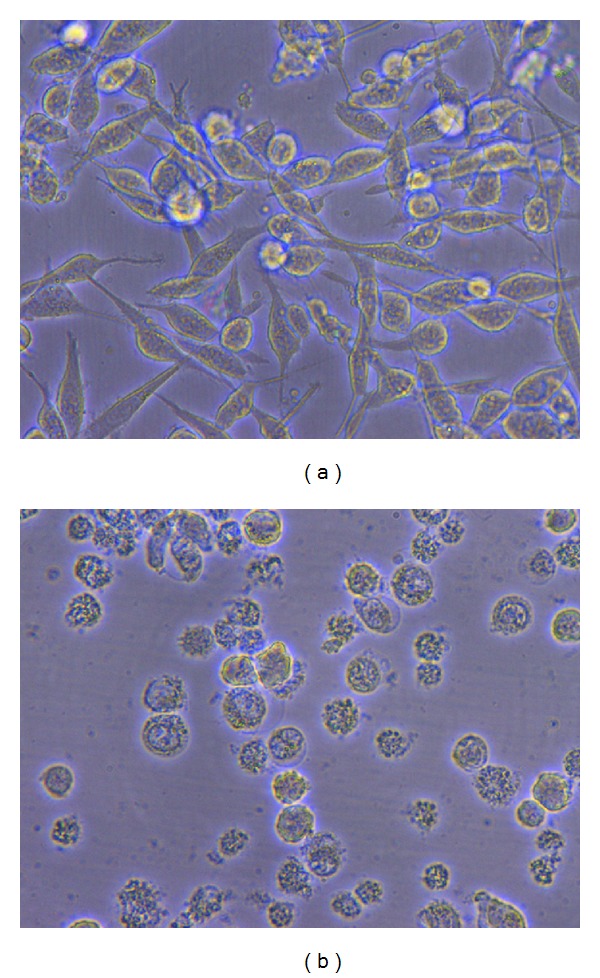
(a) Spindle-like natural and live WEHI-164, 40x; (b) spherical apoptotic WEHI-164 cells, 40x.

Fusiform or spindle-like natural and live WEHI-164 cells (Figure 1(a)) undergo morphological changes after treatment with Taxol or methanolic extract of *Dorema glabrum* seed, and because of chromatin condensation and other changes they shrink and take a spherical shape (Figure 1(b)), characteristics of apoptotic cells. 

### 3.2. MTT Test

**Figure 2:**
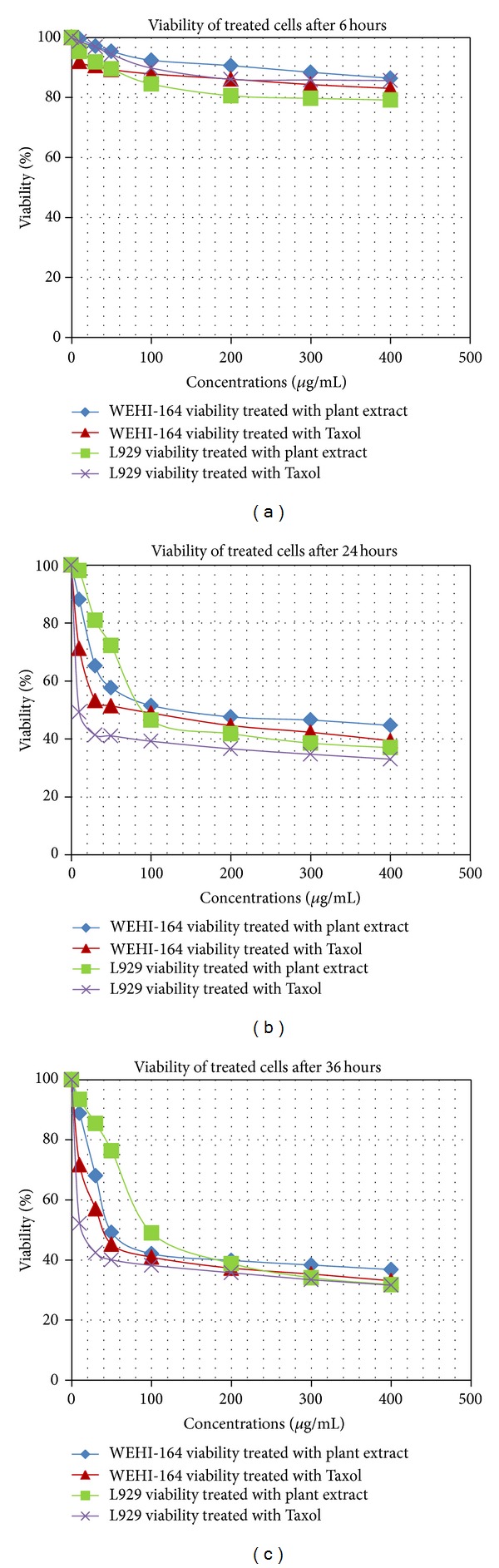
The viability of WEHI-164 and L929 cells treated with the different concentrations of plant extract in the different time periods in contrast with that of the cells treated with Taxol.

MTT assay showed a time- and dose-dependent inhibition of the cell growth by plant extract (Figure 2). As the figure shows, IC_50_ value, the concentration that causes 50% loss of cell viability, in WEHI-164 cell line is about 50 *μ*g/mL in 36 hours. By contrast the plant extract had higher IC_50_ value (about 100 *μ*g/mL in 36 hours) for normal L929 cells, meaning that it is less toxic to the normal cells than WEHI-164 cells.

Statistical analysis using independent *t*-test, which resulted in *P* < 0.0001, showed that cytotoxicity effects of 50 *μ*g/mL plant extract in 36 hours on WEHI-164 and L929 cells are significantly different.

### 3.3. Electrophoresis and Immunoblotting

**Figure 3:**
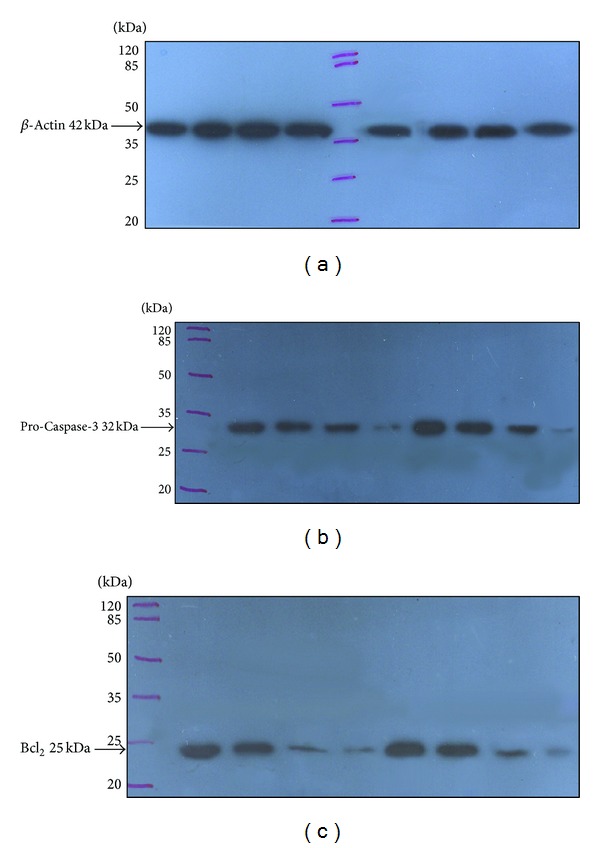
Western blot analysis of (a) *β*-actin, (b) pro-Caspase-3, and (c) Bcl_2_ production in WEHI-164 cells. From the left, first and fifth Lanes are negative controls. Lanes 2–4 and 6–8 are treated cells with different concentrations (30, 50, and 100 *μ*g/mL) of plant extract, respectively after 24 and 36 hours.

In order to compare the effects of different concentrations of plant extract on the production of Bcl_2_ and Caspase-3, all samples were unified using *β*-actin amount as an intrinsic factor. As it can be seen from Figure 3, because antizymogen Caspase-3 was used as primary antibody, in the samples related to treated cells, Caspase-3 zymogene band is weakened due to its cleavage. As mentioned previously, most caspases are synthesized as inactive zymogens and must be cleaved at two or three aspartate residues to generate the active enzyme.

The figure also shows the reduction of Bcl_2_ antiapoptotic protein in treated cells. So, both western blotting results confirmed that methanolic extract of *Dorema glabrum* seed can induce apoptosis in WEHI-164 cells.

### 3.4. Investigating bcl_**2**_ and Caspase-3 Genes Expression

**Figure 4:**
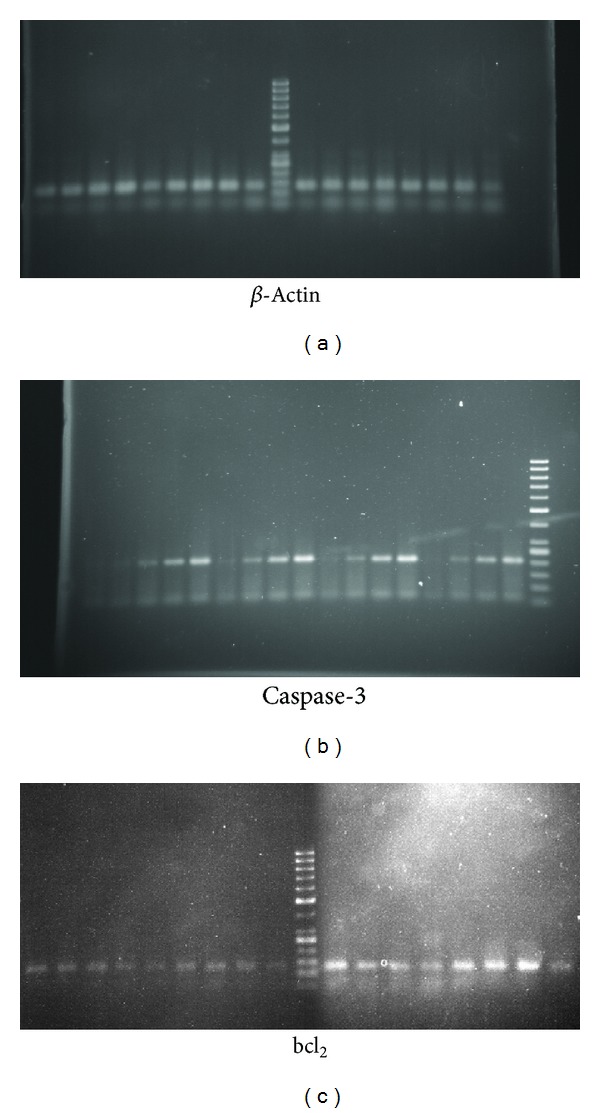
*β*-Actin (a), Caspase-3 (b), and bcl_2 _(c) gene expression. Caspase-3 gene upregulated expression and bcl_2_ downregulated expression show time- and concentration-related manner. From left, lane 1: negative control, lanes 2–5, 6–9, 10–13, and 14–17 are related to cells treated with different concentrations (10, 30, 50, and 100 *μ*g/mL of extract) after different time periods (3, 6, 12, and 24 hours). End lanes in (a) and (b), and middle lane in (c) are Ladder.

All PCR products of synthesised cDNAs were subjected to electrophoresis through 2% agarose gel followed by ethidium bromide staining in order to be visualized.  Figure 4 indicates the increasement of Caspase-3 and reduction of bcl_2_ genes expression. Again all samples were unified using *β*-actin as an intrinsic control.

### 3.5. DNA Fragmentation

**Figure 5:**
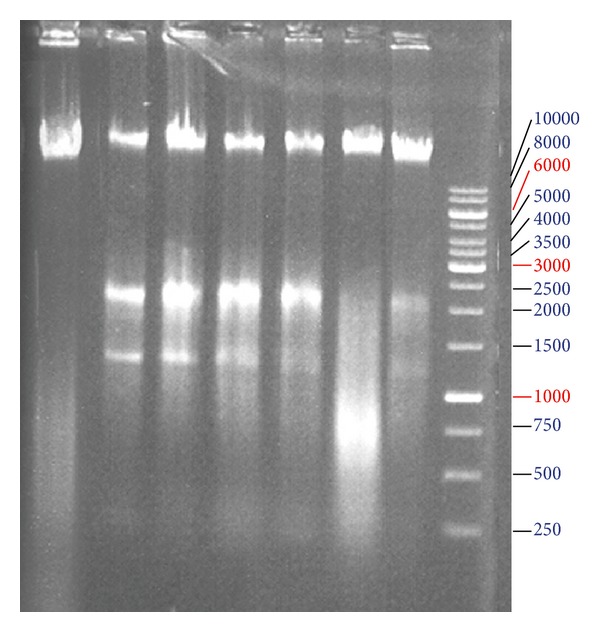
DNA ladder formation. From left, lane 1: negative control, lanes: 2–4 and treated cells with different concentrations (30, 50, and 100 *μ*g/mL) of plant extract in 24 hours, and lanes: 5–7 and treated cells in 36 hours, and lane 8 Ladder (1 kb).

DNA fragmentation can be analysed by the typical “DNA ladder” formation, for which DNA is extracted from the apoptotic cells and separated in an agarose gel. As shown in Figure 5, treatment with *Dorema glabrum* seed extract resulted in degradation of chromosomal DNA into small internucleosomal fragments, a biochemical hallmark of cells undergoing apoptosis.

## 4. Discussion

Despite a period in which pharmaceutical companies cut back their use of natural products in drug discovery, there are many promising drug candidates in the current development pipeline that are of natural origin. Technical drawbacks associated with natural product research have been lessened, and there are better opportunities to explore the biological activity of previously inaccessible sources of natural products. With the increasing acceptance that the chemical diversity of natural products is well suited to provide the core scaffolds for future drugs, there will be further developments in the use of novel natural products and chemical libraries based on natural products in drug discovery campaigns [34]. After all, traditional cytotoxic chemotherapy although kills cancer cells by indirectly inducing apoptosis unfortunately, side effects are brutal, and most tumors become resistant [17, 23, 24]. Therefore, drugs that restore the apoptotic pathways have the potential for effectively treating tumors. Herbal plants have been the basis for nearly all medicinal therapies since ancient times and constitute a common alternative for cancer prevention and treatment in many countries around the world [35]. A number of chemotherapeutic agents, with properties including apoptosis induction and antiangiogenesis, have been isolated from natural products and characterized to inhibit the development of malignancies, such as curcumin from *Curcuma longa*, epicatechin gallate from tea, paclitaxel from Pacific yew [36], Emodin, a natural anthraquinone derivative from Rheum *palmatum* L. [37] and Honokiol, a biphenyl extract from *Magnolia obovata* bark [36].

*Dorema glabrum* is a perennial medicinal plant growing in loamy or rocky slopes commonly in Armenia, Nakhichevan, and north west of Iran that is currently used as a remedy for treating cancerous diseases in folk medicine and also as a green vegetable in domestic use [30]. To evaluate the effects of *Dorema glabrum* seed extract on cell proliferation and identify its therapeutic potential, we demonstrated, for the first time, the potent cytotoxicity activity of different concentrations of methanolic extract of *Dorema glabrum* seed against WEHI-164 mouse fibrosarcoma cell line and L929 normal cell line. A successful anticancer drug should kill or incapacitate cancer cells without causing excessive damages to normal cells, meaning minimum side effects. This ideal situation is achievable by inducing apoptosis in cancer cells. Understanding the modes of action of these compounds should provide useful information for their possible applications in cancer prevention and perhaps in cancer therapy [38]. Cell cycle modulation by various natural and synthetic agents is gaining widespread attention in recent years [38]. So, multiple techniques were used to assess the antiproliferative and apoptotic effects of *D. glabrum *seed methanolic extract on cancer cells. Usually cells undergoing apoptosis display a very similar pattern of morphological changes. These include blebbing, loss of cell membarane symmetry and attachment, cell shrinkage, nuclear fragmentation, and chromatin condensation [35].

In the present study, MTT assay was performed which showed that the methanolic extract of *Dorema glabrum* seed caused growth inhibition in the WEHI-164 cells in a dose- and time-dependent manner. But it appeared less toxic in low concentrations to normal or nonmalignant cells in vitro because IC_50_ value of the plant extract for WEHI-164 cells is 50 *μ*g/mL and for L929 cells is 100 *μ*g/mL in 36 hours. This claim was confirmed by statistical analysis using independent *t*-test that resulted in *P* < 0.0001, meaning that the mean differences of cytotoxicity effects of 50 *μ*g/mL plant extract in 36 hours on WEHI-164 and L929 cells are significant. Thirty-sixh hours treatment was selected because in shorter times higher concentrations of plant extract were needed to cause 50% loss of cell viability. Since concentrations more than 50 *μ*g/mL affect L929 cells viability too, it is prefered to choose 36-hour treatment with 50 *μ*g/mL plant extract in order to avoid massive damages to normal cells. Also, we compared the effects of plant extract with the effects of Taxol, an anticancer and apoptosis inducer drug, and it should be mentioned here that the effects of plant extract on both cell lines followed the same pattern as Taxol effects on the cells (Figure 2).

Microscopic studies showed morphological changes of the cells too. Chromatin condensation, cell shrinkage, and other alterations, characteristics of apoptotic cells, cause the morphology of treated cells with the plant extract, change from spindle like to spherical shape, and also make them lose their attachment (Figure 1). In conclusion, the plant extract induced apoptosis in treated cells and not necrosis.

Also apoptosis induction was confirmed by DNA ladder technique. Treatment with the plant extract resulted in degradation of chromosomal DNA into smaller fragments (Figure 5), a biochemical hallmark of cells undergoing apoptosis [6]. Once more, induction of apoptosis, and not necrosis, by plant extract was confirmed because electrophoresis of necrotic cells' DNA results in smear not ladder.

As it was referred, apoptosis is a consequence of a highly complex and sequential cascade of cellular events, and Caspase-3 has been implicated in the execution phase of apoptosis cleaving over 100 substrates [35]. Due to our investigation results, the immunoblotting data, since 32KD Caspase-3 precursor was decreased in time- and concentration-dependent manner, methanolic extract of *Dorema glabrum* seed can induce Caspase-3 activation via its proteolytic cleavage into active subunits which enact the final irreversible commitment to death. Also from the immunoblotting results, the decrease in the amount of antiapoptotic Bcl_2_ protein is clear in the cells exposed to plant extract (Figure 3). It was mentioned that overexpression of Bcl_2_ proteins is seen in different types of tumors, which can contribute to drug-resistant state. It is believed that prevailing a blockade induced by Bcl_2_ or Bcl-xl could restore normal cellular homeostasis, reverse the drug-resistant phenotype, and restore tumor cell sensitivity to conventional chemotherapeutics [5, 20, 21].

We have also performed RT-PCR technique and demonstrated that the plant extract-dependent apoptosis was accompanied with significant increase of Caspase-3 mRNA and hence its expression and decrease of that of Bcl_2_ protein (Figure 4).

## 5. Conclusion

In conclusion, our data well established the antiproliferative effect of methanolic extract of *Dorema glabrum* seed and clearly showed that the plant extract can induce apoptosis and not necrosis in vitro, but its activities remained unknown in vivo. These results demonstrated that *Dorema glabrum* seed with antiproliferative properties, especially with IC_50_ value for cancerous cells lower than that of normal cells, might be a novel and attractive therapeutic candidate for tumor treatment in clinical practice.
